# Роль междисциплинарного ведения пациенток сo сниженным овариальным резервом, преждевременной недостаточностью яичников и нарушениями настроения

**DOI:** 10.14341/probl13576

**Published:** 2025-07-22

**Authors:** Ю. С. Абсатарова, Ю. С. Евсеева, Т. А. Зеленкова-Захарчук, Е. Н. Андреева, Е. В. Шереметьева, О. Р. Григорян

**Affiliations:** Национальный медицинский исследовательский центр эндокринологии им. академика И.И. Дедова; Национальный медицинский исследовательский центр эндокринологии им. академика И.И. Дедова; Национальный медицинский исследовательский центр эндокринологии им. академика И.И. Дедова; Национальный медицинский исследовательский центр эндокринологии им. академика И.И. Дедова; Российский университет медицины; Национальный медицинский исследовательский центр эндокринологии им. академика И.И. Дедова; Национальный медицинский исследовательский центр эндокринологии им. академика И.И. Дедова

**Keywords:** преждевременная недостаточность яичников, снижение овариального резерва, депрессия, тревога, расстройства настроения, антимюллеров гормон, беременность

## Abstract

Снижение овариального резерва и преждевременная недостаточность яичников (ПНЯ) — патологии, ассоциированные с неблагоприятным репродуктивным прогнозом, дефицитом эстрогенов и развитием множественных осложнений гипоэстрогении. В настоящее время наиболее вероятным способом реализации репродуктивных планов у данной категории пациенток является использование донорской яйцеклетки. Однако при ведении этих женщин не всегда принимается во внимание их истощенное психическое состояние, усугубляющее основное заболевание. В данной публикации представлены 2 клинических случая пациенток со сниженным овариальным резервом и ПНЯ, желающих забеременеть, с сопутствующими нарушениями настроения. Обе больные проходили лечение и динамическое наблюдение у психотерапевта с положительным результатом — самостоятельно наступила беременность. Кроме того, состояние пациентки, представленной во втором клиническом описании, отягощено патологией углеводного обмена. Представленные истории болезни иллюстрируют важность междисциплинарного подхода к ведению пациенток с преждевременной овариальной недостаточностью для повышения эффективности лечения и восстановления репродуктивной функции.

В данной публикации представлены 2 клинических случая пациенток со сниженным овариальным резервом и ПНЯ, желающих забеременеть, с сопутствующими нарушениями настроения. Обе больные проходили лечение и динамическое наблюдение у психотерапевта с положительным результатом - самостоятельно наступила беременность. Кроме того, состояние пациентки, представленной во втором клиническом описании, отягощено и патологией углеводного обмена. Представленные истории болезни иллюстрируют важность междисциплинарного подхода к ведению пациенток с преждевременной овариальной недостаточностью для повышения эффективности лечения и восстановления репродуктивной функции.

## Abstract (en)

Diminished ovarian reserve and premature ovarian insufficiency (POI) are the pathologies associated with poor reproductive prognosis, decreased estrogen production, and the development of multiple complications of hypoestrogenism. Currently, in the absence of a response to superovulation stimulation, the most likely way for patients with these conditions to achieve pregnancy is through the use of a donor oocyte. In addition, the condition of the patient presented in the second clinical description is aggravated the pathology of carbohydrate metabolism. However, the management of these women often overlooks their mental exhausting which exacerbates the underlying condition. In this publication, we present descriptions of patients with diminished ovarian reserve and POI with concomitant mood disorders who wish to become pregnant. Both patients were treated under dynamic monitoring by a psychotherapist with positive results. Moreover, spontaneous pregnancy occurred in both women described in this article. These clinical cases highlight the importance of a multidisciplinary approach in managing women with ovarian insufficiency to enhance treatment effectiveness and restore reproductive function.

## АКТУАЛЬНОСТЬ

Снижение овариального резерва и преждевременная недостаточность яичников (ПНЯ) — это актуальные медико-социальные проблемы мирового научного сообщества гинекологов-эндокринологов. К сожалению, в настоящее время риск преждевременного истощения овариальной функции и ранней менопаузы у женщин во всем мире возрастает. Согласно последним клиническим рекомендациям 2024 г. Европейского общества репродукции человека и эмбриологии (ESHRE), диагноз ПНЯ ставится на основании наличия нарушений менструального цикла в виде аменореи или олигоменореи продолжительностью не менее 4 месяцев у пациенток в возрасте до 40 лет и повышенного уровня фолликулостимулирующего гормона (ФСГ) более 25 МЕ/л, определяемого дважды с интервалом не менее месяца [1]. Распространенность овариальной недостаточности в репродуктивном возрасте в мире в среднем составляет 3,5% [2]. Общемировые диагностические критерии снижения овариального резерва до конца не разработаны, при этом данное состояние не включает в себя развитие гипогонадизма, а проявляется уменьшением количества антральных фолликулов менее 5–7 в обоих яичниках и снижением антимюллерова гормона (АМГ) менее 1,1 нг/мл [3]. Снижение овариального резерва в репродуктивном возрасте следует рассматривать как «предстадию» ПНЯ. Также существует понятие оккультной или скрытой формы преждевременного старения яичников, предложенное в 1988 г. и характеризующееся бесплодием, регулярными менструациями и умеренно повышенным уровнем ФСГ. Интересно отметить, что наступление беременности у данной категории пациенток возможно, несмотря на повышение ФСГ, в связи с периодически возникающими овуляциями на фоне стимуляции собственными гонадотропинами остаточной функции яичников [4].

Симптомы эстрогендефицита вследствие гипофункции женских половых желез значительно снижают качество жизни женщин. Помимо дискомфортных и порой невыносимых симптомов в виде приливов, ночной потливости, нарушений сна и сухости влагалища, уменьшение секреции половых стероидов приводит к снижению минеральной плотности костной ткани и неблагоприятному воздействию на сердечно-сосудистую систему. Научные исследования свидетельствуют о том, что у пациенток с преждевременной овариальной недостаточностью риск сердечно-сосудистых заболеваний и остеопороза выше, чем у женщин со своевременной менопаузой. Более того, кроме вышеуказанных последствий, ПНЯ также негативно влияет на психическое здоровье больных, значительно снижая их работоспособность и повышая риск развития тревожных и депрессивных расстройств [5]. Проблемы, связанные с бесплодием и нерегулярными менструациями, часто являются приоритетными жалобами на приеме у гинеколога, в то время как психическое здоровье непреднамеренно игнорируется. Согласно исследованию C. Menezes и соавт., у трети женщин с ПНЯ и тревогой или депрессией средней и тяжелой степени диагноз выставляется несвоевременно [6]. Причинами обращения к врачам пациенток со сниженным овариальным резервом являются бесплодие и неудачные попытки беременности. Медицинские осмотры, хирургические процедуры, финансовые расходы, неопределенные результаты лечения и другие факторы способствуют развитию психогенных тревожных реакций, серьезно снижая адаптационные возможности больных [7]. Поэтому крайне важно сосредоточиться на комплексном подходе, обязательно включая при необходимости консультацию врача-психотерапевта для осуществления медикаментозной коррекции симптомов психопатологии.

В статье описано клиническое наблюдение двух пациенток с ПНЯ, снижением овариального резерва и спонтанным наступлением беременности.

## ОПИСАНИЕ СЛУЧАЕВ

## ПАЦИЕНТКА 1

Пациентка Н., 37 лет, обратилась в отделение эндокринной гинекологии ГНЦ РФ ФГБУ «НМИЦ эндокринологии» МЗ РФ в феврале 2024 г. с жалобами на привычное невынашивание беременности, повышенную тревожность, увеличение веса c 65 кг до 107 при росте 165 см. Из анамнеза известно, что менструации начались в 13 лет, установились через 6 месяцев, продолжительностью по 5 дней через 24–25 дней, были обильные и болезненные. С 2021 г. пациентка отмечала значительное уменьшение обильности менструаций. В 2014 г. в возрасте 27 лет наступила самостоятельная беременность, завершившаяся срочными родами через естественные родовые пути. Далее в 2020 г. произошел выкидыш на 18-й неделе вследствие истмико-цервикальной недостаточности, а в 2021 г. — замершая беременность в 6 недель. В том же 2021 г. у пациентки впервые было обнаружено снижение уровня АМГ до 1,5 нг/мл.

В 2022 г. проведена лапароскопическая цистэктомия (эндометриоидная киста), иссечение очагов наружного генитального эндометриоза и раздельное диагностическое выскабливание (РДВ). По данным хромосальпингоскопии, маточные трубы проходимы. Было получено патоморфологическое заключение: эндометриома, очаги неатипической гиперплазии эндометрия.

Из анамнеза известно, что за первую беременность женщина прибавила в весе 20 кг к исходным 65 кг и далее отмечала постепенный набор массы тела. По рекомендации врача-эндокринолога, она получала лираглутид 1,8 мг с последующим снижением массы тела на 9 кг в 2022 г., в 2023 г. назначен метформин 1000 мг, однако прием препарата был нерегулярный. Индекс массы тела (ИМТ) на момент обращения в ГНЦ РФ ФГБУ «НМИЦ эндокринологии» МЗ РФ составил 39,3 кг/м².

В 2023 г. наступила самостоятельная беременность, которая прервалась на сроке в 7 недель. При обследовании установлен нормальный женский кариотип (46ХХ). Кроме того, было проведено 2 протокола стимуляции суперовуляции в 2022 г. и 2023 г., но получить качественные яйцеклетки не удалось.

По данным гормонального анализа (январь 2024 г.), ФСГ составил 25,6 Ед/л, лютеинизирующий гормон (ЛГ) — 19,8 Ед/л. В отделении эндокринной гинекологии проведено расширенное лабораторное исследование, представленное в таблице 1. Обращают на себя внимание умеренно повышенный показатель ФСГ, низкий уровень АМГ, высоконормальное значение глюкозы через 2 часа после нагрузки в ходе перорального глюкозотолерантного теста (ПГТТ) на фоне отмены в течение 10 дней сахароснижающей терапии, повышение индекса инсулинорезистентности HOMA-IR, методом высокоэффективной жидкостной хромато-масс-спектрометрии исследованы мультистероидный и эстрогеновый профиль, признаков гипогонадизма не выявлено. При проведении ультразвукового исследования (УЗИ) органов малого таза отмечено 2 антральных фолликула в обоих яичниках, выявлены сонографические признаки аденомиоза и спаечного процесса в малом тазу.

**Table:** Таблица 1. Лабораторные показатели пациентки Н.

Показатель	Результат	Единицы измерения	Референсные значения
Глюкоза натощак	5,7	ммоль/л	3,1–6,1
Глюкоза через 2 часа после нагрузки	7,7	ммоль/л	<7,8
Инсулин натощак	30,98	мкЕ/мл	2,6–24,9
Индекс HOMA-IR	7,8		
ЛГ	5,21	Ед/л	2,6–12,1
ФСГ	13,7	Ед/л	1,9–11,7
Пролактин	199,9	мЕд/л	94–500
Пролактин биоактивный	124	мЕд/л	64–365
АМГ	0,17	нг/мл	0,2–7,5
17-ОН-прогестерон	0,3	нмоль/л	0,2–6,0
11-дезоксикортизол	0,4	нмоль/л	0,0–10,0
21-дезоксикортизол	<0,10	нмоль/л	0,00–1,20
Андростендион	1,83	нмоль/л	0,80–9,00
Кортикостерон	5,3	нмоль/л	1,0–50,0
Тестостерон	0,30	нмоль/л	0,30–2,50
Кортизол (кровь), утро	249	нмоль/л	140–630
Альдостерон	509,48	пмоль/л	70,00–980,00В положении сидя
Дезоксикортикостерон	<0,15	нмоль/л	0,00–0,58
Кортизон	40	нмоль/л	33–97
Прегненолон	1,2	нмоль/л	0,0–7,0
Прогестерон	0,5	нмоль/л	0,0–1,5
Дегидроэпиандростерон	16,8	нмоль/л	4,0–50,0
17-гидроксипрегненолон	3,7	нмоль/л	0,0–20,0
11-дезоксикортизол	0,4	нмоль/л	0,0–10,0
21-дезоксикортизол	<0,10	нмоль/л	0,00–1,20
Андростендион	1,83	нмоль/л	0,80–9,00
Кортикостерон	5,3	нмоль/л	1,0–50,0
Тестостерон	0,30	нмоль/л	0,30–2,50
Кортизол (кровь), утро	249	нмоль/л	140–630
Эстрон	123	пмоль/л	0–555
Эстрадиол	157	пмоль/л	110–370

Учитывая жалобы на тревожность, пациентке была рекомендована консультация врача-психотерапевта. При осмотре выявлены: тревога ожидания очередной неудачной беременности, неустойчивое настроение с грустью, безрадостностью, дневной сонливостью, нехваткой сил для осуществления физической активности, переедание (сладости, пирожные, торты) на фоне стрессов большими объемами вечером, увеличение веса. Выставлен клинический диагноз: психогенно провоцированное тревожно-депрессивное расстройство, протекающее с ангедоническими, астеническими проявлениями, расстройство пищевого поведения по типу полисиндромального переедания (стрессогенное, гедоническое, вечернее), стресс-зависимыми нарушениями функции репродуктивной системы. Назначена терапия препаратами из группы антидепрессантов (венлафаксин до 150 мг в день) и анксиолитиков (алпразолам 1 мг ¼ табл. по состоянию при тревоге и стрессах) под динамическим контролем психотерапевта, рекомендована когнитивно-поведенческая психотерапия в режиме самотренинга и осознанного стиля питания по дробности и порционности. Лечение переносилось хорошо, в динамике отмечалось улучшение настроения, уменьшение тревоги, снижение переедания, появление больше сил.

Несмотря на рекомендации по контрацепции, спустя 3 месяца пациентка повторно обратилась к гинекологу в связи с самостоятельно наступившей беременностью сроком 13 недель, была осмотрена психотерапевтом с целью коррекции терапии. Отмечалась положительная динамика в виде нормализации настроения, редукции тревоги, переедания, снижения массы тела на 4 кг. Венлафаксин отменен на 20 неделе гестации. При проведении ПГТТ на 24 неделе глюкоза натощак — 4,9 ммоль/л, через час после нагрузки — 9,4 ммоль/л, через 2 часа — 8,4 ммоль/л. За весь период гестации зарегистрирована прибавка веса 5 кг (масса тела на момент родов — 108 кг, ИМТ — 40 кг/м²). Беременность протекала без особенностей и завершилась срочными родами через естественные родовые пути на 39-й неделе. Родилась здоровая девочка 3390 г, 50 см, по шкале Апгар 8/9 баллов.

## ПАЦИЕНТКА 2

Пациентка А. в возрасте 36 лет обратилась к гинекологу-эндокринологу в ГНЦ РФ ФГБУ «НМИЦ эндокринологии» МЗ РФ в 2023 г. с жалобами на избыточный рост волос на лице, отсутствие беременности в течение трех лет при регулярной половой жизни без предохранения, избыточный вес. Из анамнеза известно, что менструации начались в 12 лет, установились сразу, продолжительностью по 5 дней через 28–30 дней, умеренные, безболезненные. В 2018 г. пациентка была экстренно госпитализирована в стационар с маточным кровотечением, продолжавшимся 20 дней. По показаниям проведена гистероскопия с раздельным диагностическим выскабливанием. По данным патоморфологического исследования обнаружен железисто-фиброзный полип.

Также пациентка отмечает избыточную массу тела с рождения (вес при рождении — 4550 г.). Наследственность отягощена наличием сахарного диабета 2 типа у мамы и у бабушки по материнской линии. Попытки по снижению веса (диета, физические нагрузки) были без значимого эффекта. Лекарственные препараты для лечения ожирения не принимала. В 2015 г. в возрасте 28 лет во время госпитализации в стационар при обследовании выявлен сахарный диабет 2 типа на фоне экзогенно-конституционального ожирения II степени (максимальный вес — 90 кг, рост — 157 см, ИМТ — 36,5 кг/м²), инициирована сахароснижающая терапия (метформин 1000 мг в сутки). В 2020 г. к терапии добавлен эмпаглифлозин 10 мг в день. На момент приема у гинеколога-эндокринолога пациентка отметила, что сахароснижающую терапию длительное время не принимала в связи с нормальными показателями гликемии по данным нерегулярного самостоятельного измерения глюкометром. ИМТ на момент обращения составил 35,6 кг/м². Гирсутное число при осмотре — 8 баллов по шкале Ферримана-Галлвея.

С 2022 г. обнаружено повышение пролактина максимально до 800 мЕд/л. Учитывая длительный анамнез гиперпролактинемии неясного генеза, госпитализирована в 2023 г. в ГНЦ РФ ФГБУ «НМИЦ эндокринологии» МЗ РФ с целью дообследования, уточнения диагноза и определения дальнейшей тактики лечения. В ходе госпитализации были исключены эндокринные причины ожирения, бесплодия и гирсутизма. По результатам магнитно-резонансной томографии головного мозга данных за наличие очаговых и диффузных изменений не получено. Гликированный гемоглобин составил 5,9%. Выполнен ПГТТ: гликемия натощак была 5,6 ммоль/л, через 2 часа после нагрузки —11,0 ммоль/л.

По данным проведенного УЗИ органов малого таза выявлены сонографические признаки диффузной формы аденомиоза, спаечного процесса в малом тазу, по 3 антральных фолликула в обоих яичниках, варикозное расширение вен малого таза. Было обнаружено выраженное снижение АМГ до 0,7 нг/мл.

Выставлен клинический диагноз: снижение овариального резерва. Нарушенная толерантность к глюкозе. Ожирение II степени. Рекомендована модификация образа жизни, гипокалорийная диета, регулярные физические нагрузки. Назначен метформин в дозе 2000 мг.

Учитывая жалобы на тревожность, пациентке была рекомендована консультация врача-психотерапевта. При осмотре выявлены: пониженное настроение с апатией, безрадостностью, снижением стрессоустойчивости, недовольство внешним видом в лишнем весе, тревога со склонностью к накручиванию, чрезмерная двигательная суетливость на фоне стрессов, повышенная раздражительность, переедание вкусной едой (сладости) на фоне стрессов, вечером для успокоения после работы, увеличение веса, нарушение сна с пробуждениями, приемы пищи (конфеты) среди ночи. Психотерапевтом диагностированы: циклотимия у гипертимной личности, протекающая с витальными, тревожными, соматовегетативными проявлениями, расстройство пищевого поведения по типу полисиндромального (стрессогенное, гедоническое, вечернее, ночное) переедания. Назначена психофармакотерапия антидепрессантами (венлафаксин до 225 мг в день, тразодон до 150 мг на ночь, анксиолитиком (клоназепам 2 мг по ¼ табл. в минимальных дозировках ситуационно при тревоге, переедании на фоне стрессов) с рекомендацией когнитивно-поведенческой психотерапии в режиме самотренинга и осознанного стиля питания по дробности и порционности, а также индивидуальная психотерапия по запросу сепарации от матери, формирования границ личности, создания семьи и рождения ребенка. В течение последующих нескольких месяцев пациентка регулярно наблюдалась у психотерапевта, посещала индивидуальные психотерапевтические сессии по краткосрочной психоаналитически-ориентированной психотерапии. Психофармакотерапевтическое лечение переносилось хорошо, редуцировалась апатия, появились силы для осуществления физической активности (длительная ходьба, бассейн, тренажерный зал), кроме того, на индивидуальных сессиях пациентка демонстрировала быстрые положительные изменения по психоаналитической работе над собой. На фоне лечения метформином и антидепрессантами, регулярных тренировок масса тела снизилась на 15 кг за 3 месяца (с 83 кг до 68 кг при росте 157 см, ИМТ 27,6 кг/м²).

Спустя полгода повторно обратилась к гинекологу в связи с самостоятельно наступившей беременностью сроком 7 недель. Метформин был отменен, даны рекомендации по питанию, образу жизни и динамическому наблюдению. Консультирована психотерапевтом с целью коррекции лечения антидепрессантами. Далее пациентка встала на учет в женскую консультацию. Учитывая выраженную положительную динамику в виде улучшения настроения, уменьшения раздражительности и нормализации сна, больная самостоятельно отменила терапию антидепрессантами на 8-й неделе гестации. На 29-й неделе выполнен ПГТТ: глюкоза натощак составила 5,0 ммоль/л, глюкоза через 1 час — 12,2 ммоль/л, через 2 часа — 10,3 ммоль/л, выставлен диагноз: гестационный сахарный диабет (ГСД). На фоне диеты достичь целевых показателей пациентке не удалось, в связи с чем была инициирована болюсная инсулинотерапия аналогом инсулина короткого действия (аспарт) на 34-й неделе. За весь период гестации зарегистрирована прибавка веса 21 кг, вес на момент родов — 89 кг (ИМТ — 35,6 кг/м²). На 35-й неделе у пациентки произошло излитие околоплодных вод с последующими родами через естественные родовые пути. Родился мальчик 3280 г, 52 см, 7/8 баллов по шкале Апгар. Далее ребенок был переведен в отделение реанимации и интенсивной терапии новорожденных в связи с подозрением на врожденную пневмонию, проведена эмпирическая антибиотикотерапия. Ребенок выписан домой на 21-й день жизни в удовлетворительном состоянии.

Через 2 месяца после родов пациентка обратилась на телемедицинскую консультацию к психотерапевту с жалобами на неустойчивое настроение, снижение стрессоустойчивости, тревожность в связи с невозможностью грудного вскармливания и уходом за ребенком. Диагностирован психогенно провоцированный депрессивный эпизод циклотимического уровня. С пациенткой проведен психотерапевтический сеанс, настоятельно рекомендовано продолжить терапию антидепрессантами. Кроме того, запланирован ПГТТ, в настоящее время сахароснижающую терапию не принимает в связи с нормогликемией по данным самостоятельного измерения глюкометром, вес — 69 кг (ИМТ — 27,6 кг/м²).

## ОБСУЖДЕНИЕ

Снижение овариального резерва и ПНЯ — это многофакторные заболевания. Среди основных причин яичниковой недостаточности в репродуктивном возрасте выделяют генетические (мутации гена FMR1, синдром Шерешевского-Тернера и др.), аутоиммунные с образованием антиовариальных антител (изолированно или в сочетании с хроническим тиреоидитом, болезнью Аддисона, сахарным диабетом 1 типа) и ятрогенные вследствие оперативных вмешательств в малом тазу, лучевой и химиотерапии [8]. Однако, как показывает амбулаторная практика акушеров-гинекологов, в 50–70% случаев точную причину установить не удается. Независимо от этиологии, лечение женщин осуществляется в зависимости от их репродуктивных планов. Для достижения беременности при снижении овариального резерва проводится стимуляция суперовуляции с получением собственных ооцитов. У пациенток с длительным анамнезом ПНЯ, желающих забеременеть, остается только путь экстракорпорального оплодотворения с донорской яйцеклеткой. При развитии гипергонадотропного гипогонадизма у женщин до 40 лет назначается заместительная гормональная терапия половыми стероидами с целью сохранения минеральной плотности костной ткани и профилактики сердечно-сосудистых заболеваний, ранней деменции и генитоуринарного менопаузального синдрома [1].

Конкретные причины возникновения тревожных и депрессивных расстройств при ПНЯ до конца не изучены. Развитие психопатологии может быть связано с нарушением нейроэндокринной регуляции, цитокинами и психосоциальными факторами. Гипоэстрогения непосредственно вызывает нейроэндокринные нарушения, стимулируя секрецию гонадотропинов по отрицательной обратной связи.

По данным M. Gava и соавт., дефицит эстрогенов приводит к нарушению синтеза, замедлению метаболизма и снижению активности рецепторов нейромедиаторов, таких как серотонин, дофамин, норадреналин, что может привести к расстройствам настроения у пациенток с преждевременной овариальной недостаточностью [9]. Кроме того, патогенез тревожной и депрессивной психопатологии при ПНЯ может быть связан с изменением уровня трансформирующего фактора роста-β (TGF-β) и гамма-интерферона (IFN-γ) [10].

С другой стороны, у лиц с тревожными и депрессивными расстройствами наблюдаются нарушения в иммунной системе в виде преобладания воспалительных реакций из-за чрезмерной активации гипоталамо-гипофизарно-надпочечниковой оси. Последняя может активироваться хроническими негативными эмоциями, что приводит к избыточной продукции кортизола, который подавляет пульсирующий ритм высвобождения гонадотропин-рилизинг-гормона гипоталамусом, оказывая непосредственное влияние на репродуктивную систему [11]. Следовательно, психическое истощение оказывает прямое воздействие на функционирование женских половых желез.

Более того, подтверждено, что женщины с овариальной недостаточностью более подвержены психогенным тревожно-депрессивным реакциям в связи с неопределенными перспективами наступления беременности, угрозой бесплодия. Также общественная стигматизация недостаточности яичников в репродуктивном возрасте оказывает негативное действие на самовосприятие, ухудшая общее качество жизни пациенток [12].

Стоит обратить внимание, что в рассматриваемых клинических случаях нарушения репродуктивной функции сопровождались различной психопатологией. Несмотря на органическую составляющую заболеваний женской половой системы, необходимо отметить, что любое психическое расстройство усугубляет течение основной нозологии [13]. Эти наблюдения демонстрируют важность комплексного ведения пациенток со сниженным овариальным резервом, что позволит улучшить результаты терапии и привести к желаемой цели больных, в конкретных случаях к беременности.

Связь между ПНЯ и депрессивными расстройствами, вероятно, обусловлена потерей репродуктивного потенциала и дефицитом половых гормонов. Доказано, что эстрогены оказывают нейропротективное действие. Соответственно, снижение половых стероидов неблагоприятно влияет на ментальное здоровье женщин. Несколько исследований с использованием нейровизуализации показали, что активность областей мозга, отвечающих за проявление эмоций, регулируется половыми гормонами, что подтверждает гипотезу о повышении риска расстройств настроения при преждевременной овариальной недостаточности [14][15]. Tian Y. и соавт. установили, что риск депрессивного расстройства составил: отношение шансов (ОШ)=1,61; 95% доверительный интервал (ДИ): 1,11–2,33; тревожности: ОШ=3,74; 95% ДИ: 1,78–7,87 и низкого качество жизни: ОШ=2,55; 95% ДИ: 1,63–3,97 [16].

Нельзя не обратить внимание на то, как важно сопровождение психотерапевтом таких женщин не только во время преконцепционной подготовки и периода беременности, но и после родов. Актуализация расстройств настроения повышает риски развития перинатальной депрессии. Точная патофизиология этой тяжелой психопатологии до сих пор не изучена, однако ученые предполагают, что ключевым биологическим триггером может быть быстрое снижение уровней прогестерона, эстрадиола и кортизола. Кроме того, другие гипотезы сосредоточены на нейроэндокринных изменениях, которые нарушают передачу сигналов гамма-аминомасляной кислоты и приводят к снижению уровня аллопрегнанолона [17]. По данным метаанализа Xueyan Liu и соавт. [18], куда были включены 133 313 женщин, риск перинатальной депрессии повышается в 2,4 раза в случае имевшей место гестационной депрессии (95% ДИ: 1,96–2,93), в 4,8 раза при депрессии в предыдущих беременностях (95% ДИ: 1,32–17,54) и в 3 раза при депрессии в анамнезе вне периода гестации (95% ДИ: 1,62–5,93). Обнаружено, что и ГСД повышал риск развития депрессивного расстройства после родов в 2,7 раза (95% ДИ: 1,78–4,14). Эти сведения иллюстрируют, как важно вовремя обнаружить симптомы нарушения настроения, чтобы вовремя стабилизировать состояние женщины и не допустить тяжелейшего депрессивного расстройства после родов, которое может привести к весьма плачевным последствиям как для матери, так и Для ребенка.

Кроме того, описание пациентки, представленной во втором клиническом случае, является примером недооценки влияния патологии углеводного обмена на течение беременности и родов. К сожалению, поздняя диагностика ГСД, отсутствие динамического наблюдения эндокринолога и, соответственно, несвоевременная коррекция гипергликемии могли в том числе привести к преждевременным родам на 35 неделе и последующим отягощенным течением периода новорожденности у ребенка. Стоит отметить, что во втором клиническом описании дальнейшему наблюдению детского эндокринолога подлежит и новорожденный, так как имеет отягощенный анамнез по метаболическим нарушениям.

## ЗАКЛЮЧЕНИЕ

Вышеописанные клинические случаи представляют примеры пациенток со сниженной фертильностью в совокупности с расстройствами психологического характера. Совместное ведение гинекологом-эндокринологом и психотерапевтом, назначение лекарственных препаратов из группы антидепрессантов и анксиолитиков являются основными факторами положительных результатов лечения в виде наступления самостоятельной желанной беременности. Более того, необходимо подчеркнуть влияние патологии углеводного обмена на течение беременности и угрозу преждевременных родов при декомпенсации, представленное во втором клиническом случае.

Женщины со сниженным овариальным резервом и ПНЯ представляют собой очень сложную категорию больных в связи с крайне ограниченными возможностями терапевтических вмешательств. Несомненно, на сегодняшний день эти нозологии по-прежнему остаются трудноразрешимыми и приводят к бесплодию. Однако не менее значимым является психическое состояние и общее здоровье пациенток. Представленные клинические случаи доказывают, как важно выявить психопатологию и вовремя маршрутизировать женщин к психотерапевту с последующим назначением соответствующей терапии для улучшения репродуктивного прогноза.

Немаловажным фактором благополучного течения беременности и родов является нормогликемическое состояние. Отсутствие совместного ведения акушером-гинекологом и эндокринологом, несоблюдение требований алгоритмов динамического наблюдения беременных приводят к некорректной диагностике и несвоевременному лечению ГСД, повышая риски преждевременных родов у данной категории больных.

## ДОПОЛНИТЕЛЬНАЯ ИНФОРМАЦИЯ

Источники финансирования. Работа выполнена в рамках государственного задания №123021300169-4 «Эпигенетические предикторы и метаболомная составляющая аменореи различного генеза у женщин репродуктивного возраста», 2023–2025 гг.

Конфликт интересов. Авторы декларируют отсутствие явных и потенциальных конфликтов интересов, связанных с содержанием настоящей статьи.

Участие авторов. Все авторы одобрили финальную версию статьи перед публикацией, выразили согласие нести ответственность за все аспекты работы, подразумевающую надлежащее изучение и решение вопросов, связанных с точностью или добросовестностью любой части работы.

Согласие пациента. Пациенты добровольно подписали информированные согласия на публикацию персональной медицинской информации в обезличенной форме.
